# Genomic and Transcriptomic Insights into Calcium Carbonate Biomineralization by Marine Actinobacterium *Brevibacterium linens* BS258

**DOI:** 10.3389/fmicb.2017.00602

**Published:** 2017-04-06

**Authors:** Yuying Zhu, Ning Ma, Weihua Jin, Shimei Wu, Chaomin Sun

**Affiliations:** ^1^Key Laboratory of Experimental Marine Biology, Institute of Oceanology, Chinese Academy of SciencesQingdao, China; ^2^Laboratory for Marine Biology and Biotechnology, Qingdao National Laboratory for Marine Science and TechnologyQingdao, China; ^3^College of Earth Science, University of Chinese Academy of SciencesBeijing, China; ^4^College of Biotechnology and Bioengineering, Zhejiang University of TechnologyHangzhou, China; ^5^College of Life Sciences, Qingdao UniversityQingdao, China

**Keywords:** genomic, transcriptomic, marine, actinobacterium, biomineralization, heavy metal, bioremediation

## Abstract

Calcium carbonate (CaCO_3_) biomineralization has been investigated due to its wide range of scientific and technological implications, however, the molecular mechanisms of this important geomicrobiological process are largely unknown. Here, a urease-positive marine actinobacterium *Brevibacterium linens* BS258 was demonstrated to effectively form CaCO_3_ precipitates. Surprisingly, this bacterium could also dissolve the formed CaCO_3_ with the increase of the Ca^2+^ concentration. To disclose the mechanisms of biomineralization, the genome of *B. linens* BS258 was further completely sequenced. Interestingly, the expression of three carbonic anhydrases was significantly up-regulated along with the increase of Ca^2+^ concentration and the extent of calcite dissolution. Moreover, transcriptome analyses revealed that increasing concentration of Ca^2+^ induced KEGG pathways including quorum sensing (QS) in *B. linens* BS258. Notably, most up-regulated genes related to QS were found to encode peptide/nickel ABC transporters, which suggested that nickel uptake and its associated urease stimulation were essential to boost CaCO_3_ biomineralization. Within the genome of *B. linens* BS258, there are both cadmium and lead resistance gene clusters. Therefore, the sequestration abilities of Cd^2+^ and Pb^2+^ by *B. linens* BS258 were checked. Consistently, Pb^2+^ and Cd^2+^ could be effectively sequestered with the precipitation of calcite by *B. linens* BS258. To our knowledge, this is the first study investigating the microbial CaCO_3_ biomineralization from both genomic and transcriptomic insights, which paves the way to disclose the relationships among bacterial metabolisms and the biomineralization.

## Introduction

Carbonates account for 41.9% of the total carbon on Earth and represent an important carbon reservoir (Ehrlich, 1996). At the Earth’s surface, a large fraction of the insoluble carbonate is biogenic and involves bacteria, fungi, algae, and metazoan (Gadd, 2010). A significant portion of carbon exported from the sea surface to deep waters and sediments is in the form of biogenic CaCO_3_ (Berelson et al., 2007). In a geological perspective, the importance of biogenic calcification is evident from the global existence of calcium carbonate (CaCO_3_) rocks including limestone and dolomite (Heldal et al., 2012). Therefore, biogenic production and sedimentation of calcite in the ocean have profound implications for the ocean carbon cycle and ocean acidification (Heldal et al., 2012).

Biomineralization of CaCO_3_ is a phenomenon of biologically controlled or induced mineralization of CaCO_3_, and it is also an important process resulting in the production of biominerals by a range of taxa including bacteria, archaea and eukarya (Bundeleva et al., 2012; Rodriguez-Navarro et al., 2012; Benzerara et al., 2014). Generally, biomineralization is classified as either biologically controlled mineralization (BCM) or biologically induced mineralization (BIM) (Marvasi et al., 2010). BCM of CaCO_3_ is controlled through intracellular organic matrices or vesicles, which usually takes place in eukaryotes, such as molluscs, sea urchin spines, and fish otoliths (Lowenstam and Weiner, 1989). In contrast, BIM of CaCO_3_ does not involve direct control of the biomineralization process by the organisms, and it is regulated by physiological activities and carried out in open environments (Lowenstam and Weiner, 1989; Ries et al., 2008; Rodriguez-Navarro et al., 2012). BIM occurs either passively, due to metabolically driven changes in the chemistry of the bulk solution or around the living organisms, or actively, when the organism and/or its metabolic byproducts provide nucleation sites for mineralization (Mitchell and Ferris, 2006; Rodriguez-Navarro et al., 2012). BIM of CaCO_3_ typically occurs in the presence of unicellular organisms such as bacteria and is often correlated with metabolic pathways such as photosynthesis, urea hydrolysis and sulfate reduction (Dupraz and Visscher, 2005; Braissant et al., 2007; Marvasi et al., 2010). However, the precipitation of CaCO_3_ by bacteria through urea hydrolysis is the most widely used method (Anbu et al., 2016).

Urease-based microbially induced carbonate precipitation (MICP) is brought about through the ureolytic pathway, which leads to alkalinization of the surrounding environment (Stocks-Fischer et al., 1999). In addition to 2 M of NH4^+^, 1 M of carbonate ion is formed (reaction 1). Thus, in the presence of Ca^2+^ (reaction 2), calcium carbonate (CaCO_3_) is produced (Kang et al., 2014).

$${\text{CO}\left({\text{NH}}_{2}\right)}_{2}+{2\text{H}}_{2}\text{O}\to {2\text{NH}}_{4}^{+}+{\text{CO}}_{3}^{2-}\left(\text{reaction1}\right)$$

$${\text{CO}}_{3}^{2-}+{\text{Ca}}^{2+}\to {\text{CaCO}}_{3}\left(\text{reaction2}\right)$$

Calcium carbonate precipitation by many bacterial genera has been observed in different natural environments including seawater (Wright and Oren, 2005). The ecological implications of MICP are various (Wright and Oren, 2005), and the ecological relevance of MICP has been exploited in biotechnology, with applications in the bioremediation of metal contaminated soil and groundwater (Mitchell et al., 2010; Li et al., 2015).

It is generally accepted that MICP is correlated to metabolic activities and cell surface structures (Barabesi et al., 2007). A plausible link of CaCO_3_ production to *Bacillus subtilis* genes has been reported, and this research suggested that bacterial precipitation of these minerals brings some evolutionary advantage to the microorganisms (Barabesi et al., 2007). For instance, CaCO_3_ precipitation may help to fixate excess Ca^2+^ (Obst et al., 2009) or may help bacteria to survive unfavorable conditions in a cryptobiotic state (Yoshida et al., 2010). Altogether, these studies are helpful to explain why bacterial CaCO_3_ precipitation appears to be a ubiquitous phenomenon. However, the actual roles and corresponding molecular mechanisms especially from genomic and transcriptomic insights of bacteria in carbonate mineralization are still debated.

In this study, a urease positive marine actinobacterium *B. linens* BS258 was demonstrated to form and dissolve calcite precipitation in conditions at different concentration of Ca^2+^. Genome sequencing, transcriptome profiling and other assays of *B. linens* BS258 suggested that calcite precipitation and dissolution were linked to carbonic anhydrase (CA) activities, quorum sensing (QS) and other essential energy metabolisms. Heavy metal resistance and removal abilities related to MICP by *B. linens* BS258 were further dissected. Thus, the results of this study provided novel understanding of the mechanisms involved in bacterial carbonate biomineralization and proved its potentials for metal bioremediation in the marine environment.

## Materials and Methods

### Isolation and Identification of Bacterial Strains Producing Calcite

Sediments were collected from the China Yellow Sea (118°25′E, 35°56′N) and maintained in the laboratory at 4°C. The marine bacterial strains used in this study were isolated from the sediments with dilution method described as previously (Schut et al., 1993), and cultured in marine broth 2216E (5 g/L tryptone, 1 g/L yeast extract, one liter filtered seawater, 15 g agar, pH adjusted to 7.4–7.6) at 28°C. Urease-positive strains were preliminary screened with 9219 medium containing peptone (1 g), KH_2_PO_4_ (2 g), NaCl (5 g), urea (20 g), d-glucose (1 g), phenol red (0.012 g) and agar (15 g) in one liter filtered seawater, pH 6.8 ± 0.2 (Balan et al., 2012), and the plates would turn red for urease-positive strains when incubated at 28°C. CaCO_3_-producing strains were then screened from these urease-positive strains using CaCO_3_-producing medium, after incubation at 28°C for 5 days, the bacterial colonies were checked by microscope to confirm the production of calcite. The CaCO_3_-producing medium was prepared as following: nutrient broth (3 g), NaHCO_3_ (2.12 g), NH_4_Cl (10 g), CaCl_2_ (16.65 g), urea (20 g) and agar (15 g) in one liter filtered seawater. Genomic DNA was extracted from the isolate, and PCR (polymerase chain reaction) was performed to amplify the 16S rDNA gene sequence as described previously with universal primers 27F (5′-AGAGTTTGATCCTGGCTCAG-3′) and 1492R (5′-AAGGAGGTGATCCACCC-3′) (Wu et al., 2016). To determine the phylogenetic position of the *B. linens* BS258, the 16S rDNA gene sequence was analyzed by the BLAST programs^1^. The phylogenetic tree was constructed with MEGA6.0 (Tamura et al., 2013).

*Brevibacterium linens* BS258 was firstly incubated in 2216E medium to a logarithmic phase, then 1 mL starter was inoculated in 100 mL 9219 base medium for the production of calcite. The 9219 base medium was prepared as following: 10 g peptone, 5 g yeast extract, 3.33 g CaCl_2_, 2 g CH_3_COONa, 2 g KH_2_PO_4_, 0.005 g NiSO_4_, 0.005 g MnSO_4_, 5 g NaCl, 5 g urea and 20 g d-glucose in one liter filtered seawater. After incubating at 28°C for 10 days, the bacterial cultures were centrifuged at 30 g for 4 min (Riding, 2000). The collected mineral precipitate was then washed with Milli-Q water for three times and dried at 60°C for 24 h. Dried minerals were then further analyzed by Scanning Electron Microscopy (SEM), Energy-Dispersive X-ray (EDX), Transmission Electron Microscopy (TEM) and Fourier Transform Infrared Spectroscopy (FTIR).

### SEM, TEM, and EDX Analyses of the Minerals Formed by *B. linens* BS258

Minerals precipitated by *B. linens* BS258 were mounted on double-sided carbon adhesive tape on aluminum stubs prior to examination by SEM and EDX. For SEM, particulate materials mounted on stubs were sputter coated for 5 min with gold and platinum (10 nm) using a Hitachi MC1000 Ion Sputter (Li et al., 2015). Specimens were examined using a field emission scanning electron microscope (Hitachi S-3400N) operating at an accelerating voltage of 5 kV. For EDX, uncoated samples were used, and operation was at an accelerating voltage of 15 keV for 100 s (Li et al., 2015).

Aliquots of suspensions containing untreated/treated live bacterial cells and precipitated minerals were also examined using TEM with a JEOL JEM 12000 EX (equipped with a field emission gun) at 100 kV. The cell suspension was rinsed using sterile nutrient solution or Milli-Q water, centrifuged 2 min at 7000 × *g* (Bundeleva et al., 2014). Samples were taken by immersing grids coated with a carbon film for 10 s in prepared bacterial suspension and dried several minutes at room temperature. The possible precipitated carbonate observed with TEM was further confirmed using Selected Area Electron Diffraction (SAED) (Han et al., 2014).

### Calcite Precipitation and Dissolution by *B. linens* BS258 under Different Conditions

To further investigate the calcite precipitation by *B. linens* BS258 in the plate, 5 μL overnight cultured cell suspension of *B. linens* BS258 was loaded in the center of each plate with CaCO_3_ precipitation medium supplemented with different extra concentration of CaCl_2_ (0 mM, 50 mM, 150 mM, and 500 mM). The plates were incubated at 28°C for 5 days. To study the dynamics of calcite precipitation and dissolution by *B. linens* BS258, the plates supplemented with additional 150 mM CaCl_2_ in the CaCO_3_ precipitation medium were monitored with light microscopy every 12 h. Meanwhile, the urease activities of *B. linens* BS258 at the same time points were checked as described above.

### Changes in pH and Dissolved Calcium Concentrations Over Time during Growth of *B. linens* BS258 under Different Conditions

*Brevibacterium linens* BS258 was firstly incubated in 2216E medium to a logarithmic phase, then 1 mL starter was inoculated to 50 mL modified Luria-Bertani (LB) medium (10 g/L peptone, 5 g/L yeast extract, 10 g/L NaCl and 20 g/L urea) prepared with Milli-Q water supplemented with 50, 150, and 500 mM CaCl_2_ for the production of calcite. After 5 days of incubation, the pH and concentration of Ca^2+^ in the medium were measured, respectively. The supernatant for measuring pH and the concentration of dissolved calcium was collected by centrifugation at 7000 × *g* for 2 min. And the supernatant was thoroughly digested with nitric acid and perchloric acid, and diluted with Milli-Q water for calcium concentration detection (Li et al., 2013). The dissolved calcium concentrations were measured with an inductively coupled plasma source mass spectrometer (Optima 7300 DV, PerkinElmer).

### Quantitative Real-time PCR (qRT-PCR) Analyses of Carbonic Anhydrases Expression of *B. linens* BS258 under Different Conditions

For qRT-PCR assay, the cells of *B. linens* BS258 incubated in modified LB medium amended with three different concentrations of Ca^2+^ (with additional 0, 50, 150 mM Ca^2+^) were collected respectively after 6 h incubation. Total RNAs were extracted using the RNApure Bacteria Kit (DNase I) (CWBio, China). Then the total RNAs were reverse transcribed into cDNA and the transcriptional levels of three genes of CAs were determined by qRT-PCR using Sybr Green Premix Low rox (MDbio, China) and the *QuantStudio*^TM^ *6* Flex (Thermo Fisher Scientific, America). RNA degradation and contamination was monitored on 1% agarose gels. RNA purity was checked using the NanoPhotometer^®^ spectrophotometer (IMPLEN, Westlake Village, CA, USA). RNA concentration was measured using Qubit^®^ RNA Assay Kit in Qubit^®^ 2.0 Flurometer (Life Technologies, Carlsbad, CA, USA). RNA integrity was assessed using the RNA Nano6000 Assay Kit of the Bioanalyzer 2100 system (Agilent Technologies, Santa Clara, CA, USA). 16S rDNA was used as an internal reference and the relative gene expression was calculated using the 2^-ΔΔCt^ method (Qin et al., 2015). Specific primers for CAs and 16S rDNA were designed using Primer 6.0 as shown in Supplementary Table S1. All qRT-PCR runs were conducted with three biological and three technical replicates. Specific primers for CAs and 16S rDNA were designed using Primer 6.0 as shown in Supplementary Table S1.

### Transcriptional Profiling of *B. linens* BS258 Challenged with Different Concentrations of Ca^2+^

Total RNAs of *B. linens* BS258 incubated in the modified LB medium with three different concentrations of Ca^2+^ (with additional 0, 50, 150 mM CaCl_2_) after 24 h of incubation were extracted and checked as described above. The detailed protocols of RNA-seq were described in the Supplementary Information. qRT-PCR verification of the data from transcriptional profiling was performed as described above. Five genes (A2T55_RS10820, A2T55_RS11225, A2T55_RS11230, A2T55_RS01425, A2T55_RS01430) were chosen for verification of RNA-Seq data by qRT-PCR. Total RNAs were extracted and checked as described above. Then the total RNAs were reversely transcribed into cDNA and the transcriptional levels of 5 selected genes were determined by qRT-PCR using Sybr Green Premix Low rox (MDbio, China) and the QuantStudio 6 Flex (Thermo Fisher Scientific, America). 16S rDNA was used as an internal reference and the relative gene expression was calculated using the 2^-ΔΔCt^ method (Qin et al., 2015). Specific primers were designed using Primer 6.0 as shown in Supplementary Table S3.

### Co-precipitation of Ca^2+^, Cd^2+^ and Pb^2+^ by *B. linens* BS258

A modified LB medium (10 g/L peptone, 5 g/L yeast, 10 g/L NaCl, 80 mM urea and 30 mM CaCl_2_) supplemented with different metal ions was used to investigate the remediation ability of *B. linens* BS258. Briefly, *B. linens* BS258 was incubated for 24 h with shaking at 28°C in LB medium supplemented with different concentration of CdCl_2_ (0.025, 0.05, 0.1, 0.13, and 0.16 mM), or different concentration of PbCl_2_ (1, 2, 3, 4, and 5 mM). To measure the concentrations of Cd^2+^ and Pb^2+^ at the end of the incubation, the supernatant was collected by centrifugation (13400 × *g*, 2 min), and was digested completely and diluted with Milli-Q water. The concentrations of Cd^2+^ and Pb^2+^ in the solution were measured with an inductively coupled plasma source mass spectrometer (Optima 7300 DV, PerkinElmer) (Han et al., 2014).

### Statistical Analysis

The significant differences among groups were subjected to one-way analysis of variance (one-way ANOVA) and multiple comparisons by using the SPSS 18.0 program. A statistically significance was defined in our study by *P* < 0.05 (indicated by ^∗^ in all figures) or *P* < 0.01 (indicated by ^∗∗^ in all figures).

### Nucleotide Sequence Accession Numbers

The GenBank accession number for 16S rDNA gene of *B. linens* BS258 is KU883150. The GenBank accession number for the whole genome of *B. linens* BS258 is CP014869.1. The GenBank accession numbers for three CAs are A2T55_RS07325, A2T55_RS14335, A2T55_RS17060.

## Results

### Identification of Calcite-forming Bacteria Isolated from Marine Sediments

**FIGURE 1:**
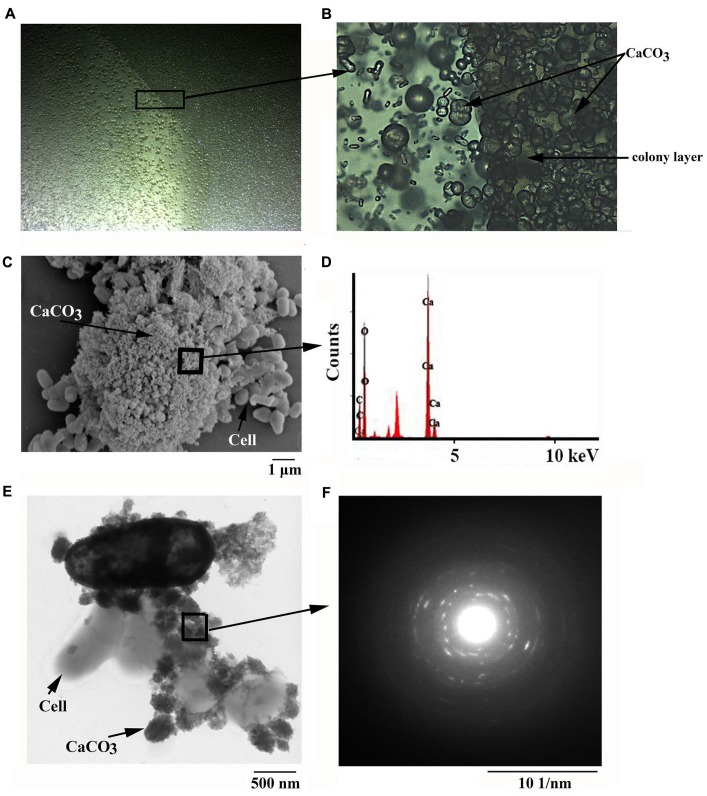
**Microscopic observation of minerals formed by *B. linens* BS258.** Light microscopic observation of minerals formed by *B. linens* BS258 with inverted microscope (Nikon TS100, Japan) at 12.5 X **(A)** or 200 X **(B)**. **(C)** Scanning electron microscopic observation of minerals precipitated by *B. linens* BS258. **(D)** Energy dispersive X-ray analysis of the selected area in **(C)**. **(E)** Transmission electron microscopic observation of minerals precipitated by *B. linens* BS258. **(F)** Electron diffraction pattern of the selected area in **(E)**. Typical images (spectra) are shown from many similar examples.

To obtain calcite-forming bacteria, ten marine sediment samples were collected from beaches along China Yellow Sea (118°25′E, 35°56′N). After incubated on isolation media, 357 growing bacteria were isolated and further screened by 9219 media to get urease-positive bacteria because calcite-forming is most often correlated with urea hydrolysis (Hammes and Verstraete, 2002). Urea in the media is hydrolyzed by urease produced by bacteria, which, in turn, leads to the increase of pH and color change in media. According to the color change in 9219 urea media, 38 isolates were identified as urease-positive, and strain 258 showed the strongest urease activity. Thereafter, urease-positive bacteria were inoculated in CaCO_3_-producing media supplemented with 330 mM urea and 150 mM CaCl_2_, and crystal-precipitating colonies were checked after 5 days cultivation with stereoscopy. Consistent with the urease activity, strain 258 was found to exhibit pronounced calcite biomineralization, which could form 1000s of crystals with different shapes and sizes both on and around the colony (**Figures 1A,B** and Supplementary Figure S1). In view of the high homology with marine actinobacteria *Brevibacterium linens* by 16S rDNA phylogenetic analysis (Supplementary Figure S2), this strain was designated as *Brevibacterium linens* BS258 and was selected for further experiments due to its obvious urease-producing and calcite-precipitating abilities.

### SEM, TEM, EDX and FTIR Analyses of the Calcite Formed by *B. linens* BS258

To get more insights of the minerals precipitated by *B. linens* BS258, the precipitates separated from 9219 base liquid media were further chosen for SEM analysis. SEM results revealed that bacteria imbedded in the crystal matrices (**Figure 1C** and Supplementary Figure S3B), which coincided with the report that the bacteria served as the nucleation sites for the mineralization process (Achal et al., 2009). In addition, imprints of the cells were also observed on the surface of the different minerals (Supplementary Figures S3C–F), which further demonstrated that bacterial cells might provide nucleation sites or structural support for the process of mineral precipitation. The small holes matched the diameters of cells of *B. linens* BS258 very well, which could also be taken as evidence for the importance of cells in carbonate biomineralization (Supplementary Figures S3A,C,D). Similar results were acquired using TEM on whole single cell and its surroundings. Representative examples of cells in the control (nutrient-rich, without adding CaCl_2_) and precipitation experiment (nutrient-rich, 30 mM CaCl_2_ was added) were shown in **Figure 1E** and Supplementary Figure S4. The cells in the nutrient media without calcium appeared intact without any traces of solid precipitates in the vicinity of the cells (Supplementary Figure S4A). In contrast, significant CaCO_3_ precipitation occurred in the vicinity of bacterial cells in the media containing calcium (**Figure 1E** and Supplementary Figures S4B–D).

To ensure that the crystals produced by *B. linens* BS258 were indeed CaCO_3_ mineral polymorphs, they were examined using EDX combined with SEM. High amount of calcium in the bacterial sample indicated that calcite was present in the form of CaCO_3_, also those spectra showed in EDX analysis indicated Ca, C, O peaks associated with calcite crystals (**Figure 1D**). Moreover, high-resolution images of the crystals from TEM and the associated SAED (**Figure 1F**) showed that they were well-crystallized calcite single crystals as described previously (Benzerara et al., 2003). Under TEM, the amorphous nature of the mineral precipitates is clearly evidenced (**Figure 1E** and Supplementary Figure S4). They start to form as thin coatings defining the cell surfaces (Supplementary Figure S4B), and evolve into globular precipitates attached to cell surfaces and within the bacterial organic film between cells (**Figure 1E** and Supplementary Figure S4C). The globules have a diameter of few 10s to few 100 nm, and show abundant electron-lucent centers resulting from devolatilization caused by electron-beam irradiation (**Figure 1F**).

Additionally, FTIR was also applied in an attempt to further characterize the crystals formed by *B. linens* BS258 (Supplementary Figure S5). The FTIR spectra showed broad bands in the high-energy region, which probably belonged to the water-related band (3374 cm^-1^), indicating the minerals were in a hydrated form. Theoretically, there are four vibrational modes for carbonate ions, ν1 (∼1000 cm^-1^), ν2 (∼ 873 cm^-1^), ν3 (1650∼1300 cm^-1^) and ν4 (∼700 cm^-1^) (Li et al., 2015). As seen in Supplementary Figure S5, there were also four peaks corresponding to ν1 (1086 cm^-1^), ν2 (876.5 cm^-1^), ν3 (1472.6 cm^-1^) and ν4 (745.5 cm^-1^) presented in the crystals formed by *B. linens* BS258, which all indicated the presence of carbonate. Combining the results of SEM, EDX, TEM, SEAD and FTIR, we concluded that the crystals formed by *B. linens* BS258 were indeed CaCO_3_.

### Calcite Precipitation and Dissolution by *B. linens* BS258

**FIGURE 2:**
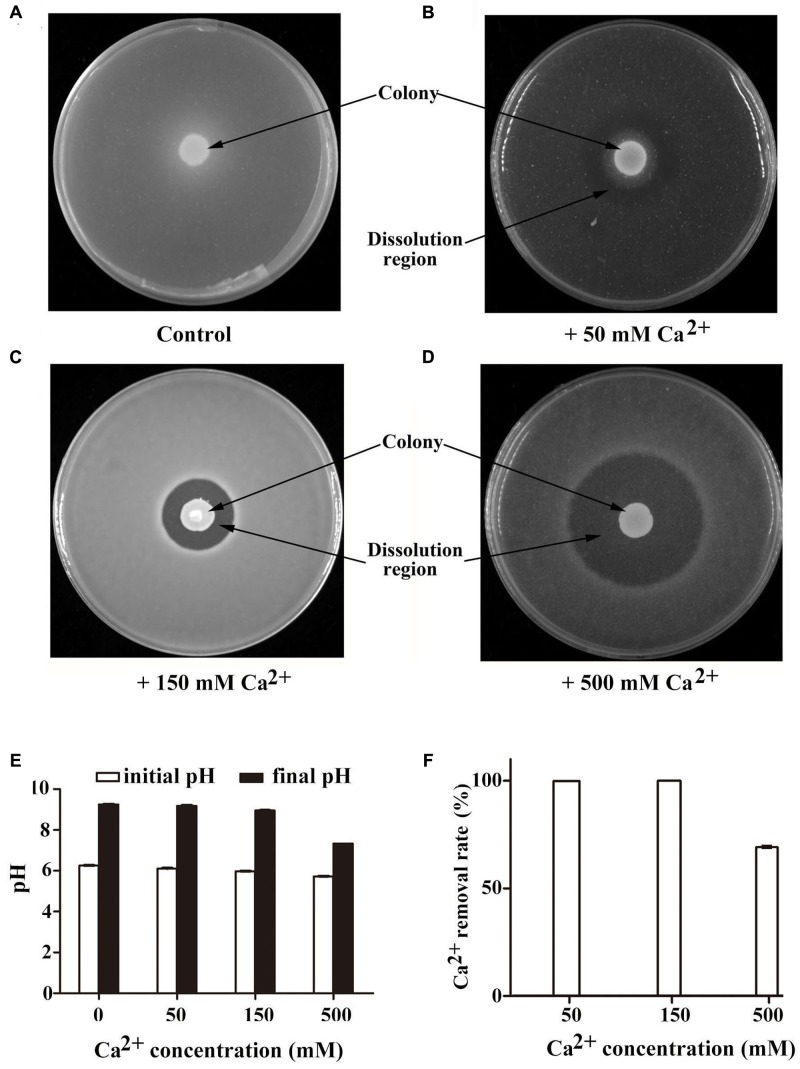
**Characterization of calcite precipitation of *B. linens* BS258 in different concentration of Ca^2+^.** Calcite precipitation and dissolution patterns in the CaCO_3_-producing media supplemented with additional 0 mM **(A)**, 50 mM **(B)**, 150 mM **(C)** or 500 mM CaCl_2_ **(D)** after growth of *B. linens* BS258 for 5 days at 28°C. Typical images are shown from many similar examples. **(E)** pH changes of BS258 in the liquid medium amended with additional 0, 50, 150, and 500 mM Ca^2+^, respectively. **(F)** Ca^2+^ removal rate of BS258 in the liquid medium amended with additional 0, 50, 150, and 500 mM Ca^2+^, respectively.

The concentration of Ca^2+^ is one of the most important factors determining the formation of calcite (Hammes and Verstraete, 2002). In order to investigate the effects of Ca^2+^ concentration on the mineralization of *B. linens* BS258, we checked calcite formation of this strain in the media supplemented with different concentration of Ca^2+^. As expected, *B. linens* BS258 could form obvious calcite precipitation on or around the colony but not the area far from the colony in the medium without additional Ca^2+^ added (**Figure 2A**). However, this bacterium started to dissolve the formed calcite crystals around the colony when additional 50 mM Ca^2+^ was added in the medium (**Figure 2B**). More interestingly, calcite crystals around the colony were completely dissolved when additional 150 mM Ca^2+^ was added (**Figure 2C**). It is noteworthy that the size of clear zone gradually became bigger with the concentration of Ca^2+^ increased up to 500 mM in the medium (**Figure 2D**). To investigate the details of calcite precipitation and dissolution by *B. linens* BS258, the plate with 150 mM Ca^2+^ was checked by light microscopy with high magnification. No any obvious calcite precipitates around the colony were observed (Supplementary Figure S6B) even lots of crystals covered the surface of the colony completely (Supplementary Figure S6A). There was a clear boundary between the clear zone and precipitation layer in the medium, and the density in the clear region margin was even higher than that in the medium (Supplementary Figures S6C,D).

### Changes in pH and Calcium Concentration Over Time during Growth of *B. linens* BS258

To further understand the changes of pH and Ca^2+^ concentration in the medium during the process of calcite-producing, *B. linens* BS258 was incubated in modified LB medium for 5 days, and pH and Ca^2+^ concentrations were measured at the end of incubation. Correspondingly, *B. linens* BS258 induced pH increase in media supplemented with different concentrations of Ca^2+^, which was a key characteristic for CaCO_3_ bioprecipitation. Meanwhile, ΔpH (pH difference between the final and initial timepoint of incubation) of the medium declined from 2.99 to 1.61 when Ca^2+^ concentrations increased from 0 to 500 mM (**Figure 2E**), which indicates that cells produced more H^+^ with the concentration of Ca^2+^ increased and is in accordance with the results of calcite dissolution in **Figures 2A–D**. Notably, as for the media amended with 50 mM and 150 mM Ca^2+^, more than 99% Ca^2+^ was removed from the supernatant, and Ca^2+^ removal rate of the medium with 500 mM Ca^2+^ was still up to 69.2% (**Figure 2F**). Therefore, *B. linens* BS258 will be a good candidate for removal of Ca^2+^ from industrial wastewater via an effective, eco-friendly and simple method in the future.

### Genome Sequencing and Analysis of *B. linens* BS258

In order to understand the molecular mechanisms of calcite formation by *B. linens* BS258 and exploit its potentials, whole genome of *B. linens* BS258 was sequenced. The sequencing results showed that the genome of *B. linens* BS258 consisted of one circular chromosome of 3,862,244 bp (**Table 1**). Notably, six genes were found to be involved in urea catabolic process (Supplementary Figure S7), including three urease genes (urease subunit alpha, urease subunit beta, urease subunit gamma) and three urease accessory proteins (UreF, UreH, UreG). To our knowledge, this is the first *Brevibacterium* sp. with complete genome sequenced, which provides a platform to pursue the molecular mechanisms of CaCO_3_ biomineralization.

**Table 1:** General genome features of *B. linens* BS258.

Features	Value
Genome size (bp)	3,862,244
G + C content (%)	64.16
Chromosome	1
Total number of genes	3423
Protein coding genes (CDSs)	3260
rRNAs	12
tRNAs	47
Genomic island	9
Protein coding genes involved in inorganic transport and metabolism	137


### The Involvement of Carbonic Anhydrase in Calcite Dissolution

**FIGURE 3:**
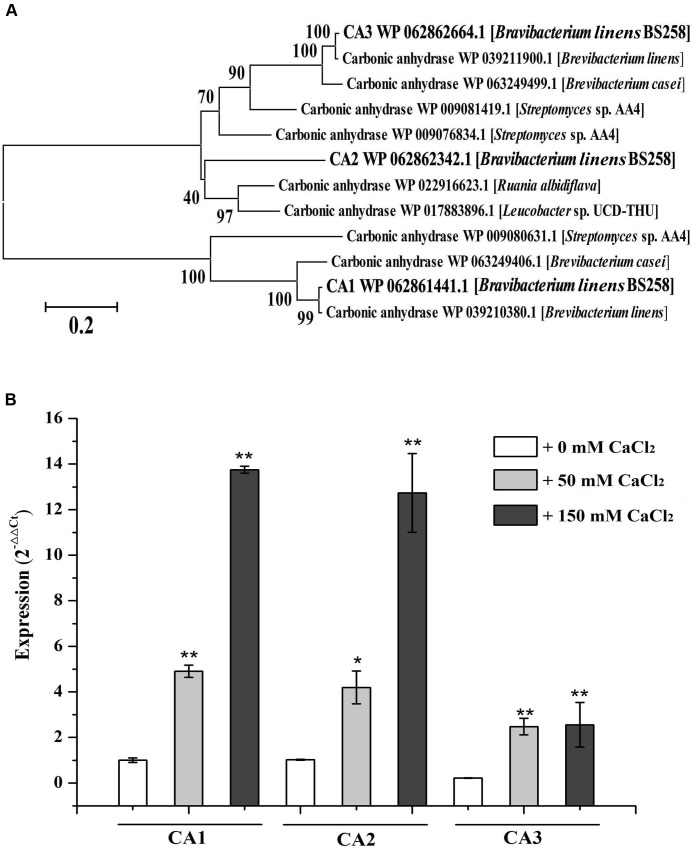
**Characterization of carbonic anhydrases of *B. linens* BS258.** **(A)** The consensus phylogenetic tree of three carbonic anhydrases of *B. linens* BS258 with other related carbonic anhydrases obtained from GenBank (accession numbers are indicated before the species name) constructed by the neighbor-joining method. Numbers above the branches are bootstrap values based on 1000 replicates. **(B)** The expression change patterns of three carbonic anhydrases of *B. linens* BS258 with the increase of Ca^2+^ concentration. All qRT-PCR runs were conducted with three biological and three technical replicates. CA: carbonic anhydrase. *P* < 0.05 indicated by *^∗^* and *P* < 0.01 indicated by ^∗∗^ versus the control (0 mM CaCl_2_) in the same group.

One of the notable characteristics of calcite precipitation by *B. linens* BS258 is that it can dissolve formed calcite when Ca^2+^ concentration changes (**Figure 2**). Notably, CA derived from *Bacillus mucilaginosus* was reported to be responsible for the wollastonite dissolution (Xiao et al., 2015). CA is capable of catalyzing the reversible hydration reaction, ${\text{CO}}_{2}+{\text{H}}_{2}\text{O}↔{\text{HCO}}_{3}^{-}+{\text{H}}^{+}$, of atmospheric and self-generated CO_2_ (Smith and Ferry, 2000; Xiao et al., 2015). There are three carbonic anhydrases (CA1, CA2, CA3) encoding genes in the genome of *B. linens* BS258, and the phylogenetic results indicates that CA3 is much closer related to CA2 than that of CA1 (**Figure 3A**). To investigate whether CA is involved in the calcite dissolution of *B. linens* BS258, qRT-PCR was performed to check the expression of CA in different conditions where dissolution happened. The results showed that the expressions of three CAs were all dramatically up-regulated when the concentration of calcium increased in the media, which was consistent well with the trend of calcite dissolution (**Figure 3B**). The consistency between the increase in CA gene expression and calcite dissolution suggests that CAs of *B. linens* BS258 play a role in the dissolution of calcite at atmospheric CO_2_ concentrations.

### Transcriptome Analyses of Calcite Precipitation in Different Concentration of Ca^2+^

**FIGURE 4:**
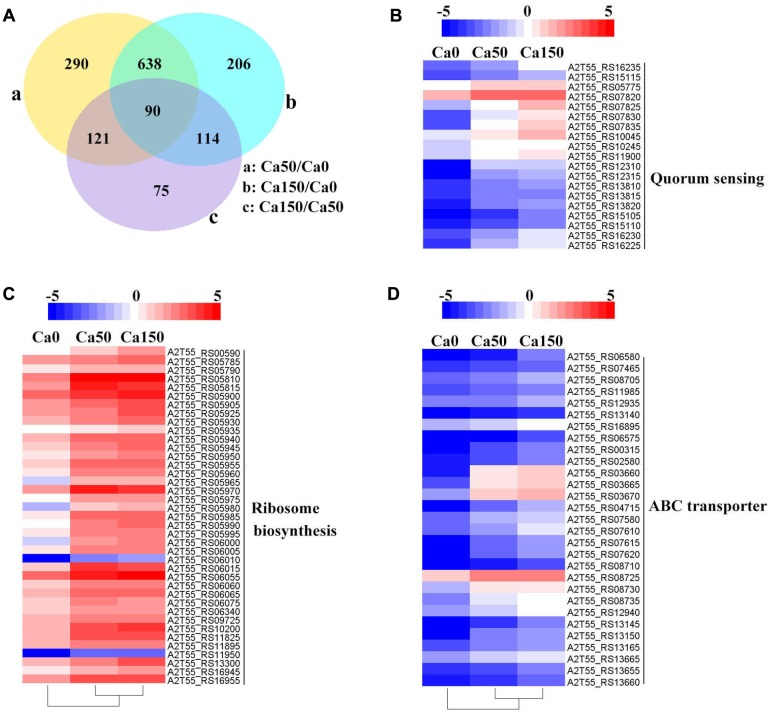
**Transcriptome profiles of *B. linens* BS258 challenged in different concentration of Ca^2+^.** **(A)** Differentially expressed genes (DEGs) that are unique or shared among three groups of Ca0 (without additional Ca^2+^ amended), Ca50 (with additional 50 mM Ca^2+^ amended) and Ca150 (with 150 additional Ca^2+^ amended). Numbers of each circle show the number of Ca^2+^ stress responsive DEGs that are uniquely (inside of non-overlapping part) or commonly (inside of overlapping part) regulated. **(B)** Heat map shows the Ca^2+^ stress responsive relatively up-regulated DEGs related to quorum sensing among Ca0, Ca50, and Ca150 enriched by KEGG analysis. **(C)** Heat map shows the Ca^2+^ stress responsive relatively up-regulated DEGs related to ribosome biosynthesis among Ca0, Ca50, and Ca150 enriched by KEGG analysis. **(D)** Heat map shows the Ca^2+^ stress responsive relatively up-regulated DEGs related to quorum sensing among Ca0, Ca50, and Ca150 enriched by KEGG analysis. All the Genbank accession numbers are provided together with the corresponding heat map. The heat map is made by the software of Heml 1.0.3.3.

Transcriptome analysis is a powerful tool for revealing the molecular mechanisms underlying the responses of bacteria to environmental stressors. To disclose the overall perspective of biomineralization of *B. linens* BS258, we investigated the transcriptome of this bacterium under different concentration of Ca^2+^. The three *B. linens* BS258 transcriptome examples were Ca0 (without additional Ca^2+^ amended), Ca50 (with additional 50 mM Ca^2+^ amended) and Ca150 (with additional 150 mM Ca^2+^ amended). A total of 1534 unigenes were differentially expressed between any two-way comparison of Ca0, Ca50 and Ca150 (| fold changes| > 2, corrected *P* < 0.005) (**Figure 4A**).

**FIGURE 5:**
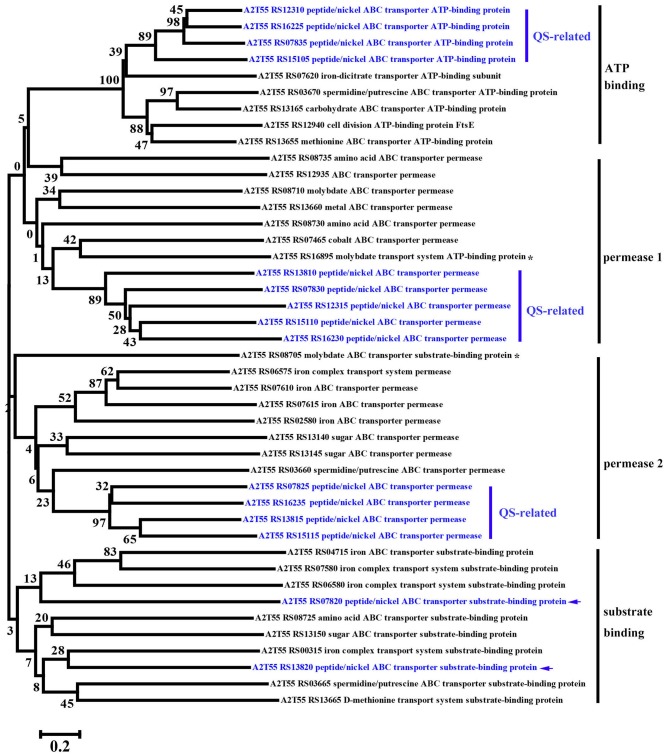
**Phylogenetic analysis of up-regulated DEGs linked to QS-related ABC transporters and other regular ABC transporters among Ca0, Ca50, and Ca150 enriched by KEGG analysis in *B. linens* BS258.** The consensus phylogenetic tree is constructed by the neighbor-joining method. Numbers above the branches are bootstrap values based on 1000 replicates. The star (^∗^) means the gene is out of the exact scope of ATP binding, permease and substrate binding counterparts of ABC transporter. The arrow indicates the position of QS-related peptide/nickel ABC transporter substrate-binding protein.

KEGG enrichment analysis showed that QS, ribosome biosynthesis, and ABC transporter were enriched among the two-way comparison of Ca0, Ca50, and Ca150 (Supplementary Tables S2A–C). Under the term of “Quorum Sensing,” expression of 19 DEGs was enhanced continuously by the increase of calcium concentration (**Figure 4B**). Notably, most up-regulated genes related to QS were found to encode peptide/nickel ABC transporters. One of the DEGs in QS was also controlled by “Fatty acid metabolism,” namely long-chain fatty acid-CoA ligase (A2T55_RS10245). Ribosome is an intracellular organelle that serves as the site of biological protein synthesis. There were 39 DEGs involved in ribosome biosynthesis that showed continuously up-regulated trend with the increase of calcium concentrations (**Figure 4C**). As the calcium concentration increased, the expression of 28 DEGs enriched in “ABC transporter” was progressively up-regulated (**Figure 4D**). The substrates transported by the ABC transporters of the 28 up-regulated DEGs were sugar, amino acid, spermidine/putrescine, metal, iron and molybdate (**Figure 5**). The DEG of ABC transporter with the highest fold change was spermidine/putrescine ABC transporter permease (A2T55_RS03660). To disclose the relationship between the QS-related transporters and other 28 DEGs enriched ABC transporters, we analyzed the phylogenetic relationship of all transporters mentioned above. Interestingly, the up-regulated QS-related transporters annotated as ATP binding protein and permease were clustered together (**Figure 5**). On the other hand, 28 DEGs enriched ABC transporters were clustered into four subgroups, ATP binding protein, two permeases and substrate binding protein (**Figure 5**), which comprise the full functional counterparts of ABC transporter (Hiron et al., 2010).

### Verification of RNA-seq Data

Reliability of the RNA-seq data was verified by qRT-PCR analysis. Totally, five genes were selected for validation. Consistently, similar trends were observed between qRT-PCR and RNA-seq results, which supported the validity of the RNA-seq data (Supplementary Figure S8).

### Heavy Metal Sequestration Assays of *B. linens* BS258

**FIGURE 6:**
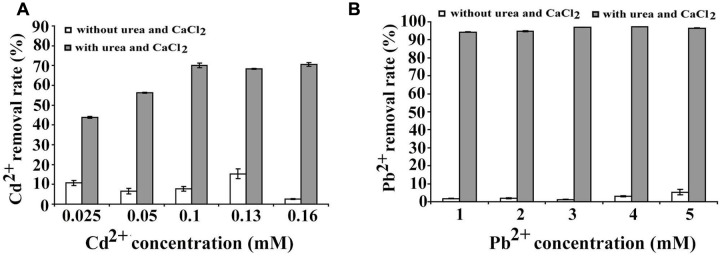
**Heavy metal ions removal by *B. linens* BS258.** **(A)** Cd^2+^ removal test by *B. linens* BS258 in different concentration of Cd^2+^ with or without amended with 30 mM Ca^2+^ and 80 mM urea in the medium. **(B)** Pb^2+^ removal test by *B. linens* BS258 in different concentration of Pb^2+^ with or without amended with 30 mM Ca^2+^ and 80 mM urea in the medium.

Microbial carbonate precipitation is known as an efficient process for the remediation of heavy metals from contaminated environments (Li et al., 2015). Next, we sought to check the capability of *B. linens* BS258 to remove heavy metals. Within the genome of *B. linens* BS258, there are both cadmium and lead resistance gene clusters. Therefore, we checked the sequestration abilities of Cd^2+^ and Pb^2+^ by *B. linens* BS258. The results showed that *B. linens* BS258 only removed very few heavy metals if Ca^2+^ and urea were absent in the media. However, heavy metal removal abilities increased dramatically if 30 mM Ca^2+^ and 80 mM urea were present in the media (**Figure 6**). Taken together, the above results indicated that *B. linens* BS258 can only remove these heavy metals by co-precipitation with Ca^2+^ by *B. linens* BS258. We proposed that the possible molecular structures of these minerals could be mixtures of CaCO_3_, XCO_3_ and (Ca_n_X_1-n_)CO_3_ (X is Cd or Pb) as described previously (Li et al., 2015). On the other hand, after incubation supplemented with different heavy metals, all media became alkaline (∼pH 9.2), which is similar to that of medium with only Ca^2+^. These results showed that supplement of heavy metal ions didn’t have obvious impact on the Ca^2+^ precipitation, while heavy metal ions could be effectively precipitated.

## Discussion

**FIGURE 7:**
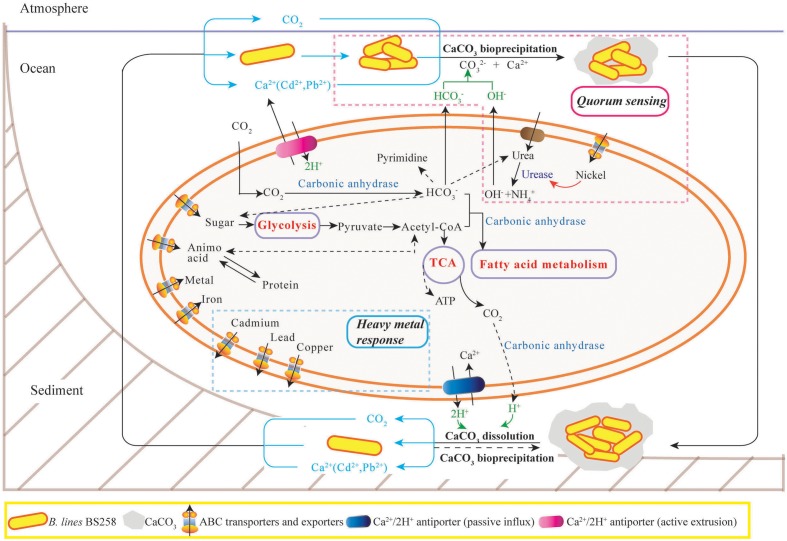
**The proposed model of calcite precipitation by *B. linens* BS258 in the ocean ecosystem.** Firstly, *B. linens* BS258 produces QS signals at a basal rate as the cells grow and divide until a threshold concentration of QS signals (like nickel concentration) has accumulated, and then the bacteria start to form calcite precipitation with the synergic action of urease, carbonic anhydrase and other necessary factors in the ocean surface. Secondly, the formed minerals precipitate to the sea floor and form the sediments. Lastly, the sediments are dissolved in the harsh conditions like high concentration heavy metal ions and the free cells go back to the ocean surface for the next round calcification. Therefore, the concentration of heavy metals in the environment is a key factor determining the equilibration between calcite precipitation and dissolution. During the course of calcite precipitation and dissolution, many energy metabolisms close related to tricarboxylic acid cycle, such as sugar biosynthesis and glycolysis, amino acid biosynthesis and degradation, fatty acid metabolism, are up-regulated. Correspondingly, the activities of many transporters of metal ions, amino acid and sugar are also positive regulated. More details are described in the Section “Discussion.” TCA: tricarboxylic acid cycle. ABC transporter: ATP-binding cassette transporters.

In this study, the marine derived actinobacterium *B. linens* BS258 was demonstrated to effectively precipitate CaCO_3_ minerals in the plates and liquid media combining the results of SEM, EDX, TEM, SAED and FTIR (**Figure 1** and Supplementary Figures S1, S3–S5). It is known that MICP is a rather straightforward chemical process governed by four key factors: (i) Ca^2+^ concentration, (ii) the concentration of dissolved inorganic carbon, (iii) the pH and (iv) the availability of nucleation sites (Hammes and Verstraete, 2002). Correspondingly, in our study, the pH of the liquid cultures (**Figure 2E**), amount of carbonate ion could be increased significantly through the ureolytic pathway of *B. linens* BS258 (**Figure 7** and Supplementary Figure S7 and reaction 1). Thus, in the environment with free Ca^2+^, CaCO_3_ could be effectively formed by using cells as nucleation sites (**Figure 1C**).

One of the important reasons for microbes to form minerals is to enhance their competitiveness in a microbial community via the detoxification of poisonous inorganic species (Ehrlich, 1996). Generally, bacteria keep tight control of their intracellular Ca^2+^ concentration at 100–300 nM because of the key role of calcium in the regulation of many essential processes and its potential detriment to cell structures (Dominguez, 2004). In this study, for *B. linens* BS258, in the medium amended with 150 mM CaCl_2_, the extracellular Ca^2+^ concentration is about 10^5^ times higher than that of intracellular, thus, passive transport usually accounts for calcium influx as previous reported (Norris et al., 1996; Hammes and Verstraete, 2002). To survive in such harsh conditions, the cells are required to actively export of intracellular excess calcium to reduce intracellular Ca^2+^ and simultaneously compensate the proton loss, which could be performed via the ATP dependent calcium pumps (**Figure 7**). With the extrusion of Ca^2+^ and uptake of protons in the same region, a localized increase in pH would happen, together with the alkalization caused by ureolytic pathway would form an ideal localized precipitation micro-environment (**Figure 7**).

Another important characteristic of *B. linens* BS258 toward calcite precipitation is that the formed calcite around the colony could be dissolved to some extent when increasing the concentration of Ca^2+^ in the media (**Figures 2** and Supplementary Figure S6). For *B. linens* BS258, the expression levels of CAs were dramatically up-regulated when higher calcium concentration existed in the medium (**Figure 3**). CA is able to catalyze 1 molar CO_2_ to 1 molar ${\text{HCO}}_{3}^{-}$ and H^+^, which suggested that the production of H^+^ would be up-regulated while calcium concentration increased. Once the concentration of H^+^ is high enough to dissolve the formed calcite precipitation around the colony, the calcite dissolution happens (**Figure 2**).

It is reported that microbes are able to dissolve a mineral by using it as a source of energy to enhance competitiveness in a microbial community (Ehrlich, 1996). CA can participate in the formation of malonyl-CoA with bicarbonate and acetyl-CoA as the substrate (Costa et al., 2007), which means CA is an important regulator of fatty acid metabolism. Additionally, the dissolution of calcite generated a certain amount of ${\text{HCO}}_{3}^{-}$ by CO_2_ hydration, which is a key substrate for many essential biological pathways including gluconeogenesis, lipogenesis, ureagenesis and synthesis of several amino acids (**Figure 7**) (Xiao et al., 2015). For *B. linens* BS258, the expressions of three CAs were all markedly up regulated with the increase of Ca^2+^ concentration (**Figure 3B**), which is in accordance with the suggestion that an increase in CA gene expression level is advantageous to the microbe’s survival chances in the harsh environments (Xiao et al., 2015).

To better understand the calcification of *B. linens* BS258, the whole genome of this bacterium was sequenced and the transcriptome profiling of *B. linens* BS258 challenged by different condition of Ca^2+^ was further performed. Surprisingly, KEGG enrichment analysis showed that only three pathways related to QS, ribosome biosynthesis, and ABC transporter were enriched (**Figure 4**). With the increase of Ca^2+^ concentration, the expression of ribosome biosynthesis and ABC transporter in charge of sugar and amino acid transport was significantly up-regulated (**Figures 4C,D**), which guarantees *B. linens* BS258 to reach higher microbial density. Consistently, the expression of genes related to QS was also markedly up-regulated (**Figure 4B**). It is reported that QS enhancement of stress tolerance, therefore having a broad impact in bacterial ecology (Garcia-Contreras et al., 2015). Our transcriptome data indicate that *B. linens* BS258 changes regulation of its biochemical pathways in a manner that is adaptive for a cooperative lifestyle in the presence of calcium to obtain phytoplankton-derived nutrients and higher microbial density.

It is noteworthy that most up-regulated genes related to QS were all peptide/nickel ABC transporters counterparts (Supplementary Table S2B). Interestingly, in bacteria, nickel uptake is mediated by peptide/nickel ABC transporters (Hiron et al., 2010). As soluble Ni^2+^ in natural environments usually exists in trace amounts, high-affinity uptake of nickel is needed to assure intracellular metalloenzyme activities (Hiron et al., 2010). It is well known that urease is a multimeric nickel-dependent enzyme mainly involved in the neutralization of acidic environments and influence the MICP to a great extent (Achal et al., 2009), and the activity of urease was determined by nickel transporters (Hiron et al., 2010). Hiron et al. (2010) found that a nickel ABC-transporter of *Staphylococcus aureus* was involved in urinary tract infection by forming infection stones through ureolytic MICP. Therefore, it is understandable that the expressions of QS-related peptide/nickel ABC transporters in *B. linens* BS258 were all evidently up-regulated with the increase of Ca^2+^ concentration. More functions of QS and its related peptide/nickel ABC transporters need to investigate further and eventually disclose the full perspective of biomineralization as illustrated in **Figure 7**.

The ecological relevance of MICP has been exploited in biotechnology with applications in many fields including bioremediation of heavy metal contamination (Li et al., 2015). In our experiments, *B. linens* BS258, incubated in media supplemented with urea and different metal ions showed very good metal removal abilities through the co-precipitation with calcium (**Figure 6**), which indicates that this bacterium is powerful and promising in the application of heavy metal pollution management.

## Author Contributions

YZ, NM, and CS conceived and designed the experiments. YZ finished the calcite production-bacteria screening and genome sequencing. NM performed the transcriptome analyses and heavy metal removal experiments. WJ helped to do the FTIR analysis. YZ, NM, and CS analyzed the data. YZ, NM, and CS prepared the figures and wrote the paper. SW helped to write the manuscript. All authors reviewed the manuscript.

## Conflict of Interest Statement

The authors declare that the research was conducted in the absence of any commercial or financial relationships that could be construed as a potential conflict of interest.

## Footnotes

1 http://blast.ncbi.nlm.nih.gov/Blast.cgi
