# Supplementary material for: Genomic and Transcriptomic Insights into Calcium Carbonate Biomineralization by Marine Actinobacterium Brevibacterium linens BS258
Source: Front Microbiol. 2017 Apr 6;8:602. doi: 10.3389/fmicb.2017.00602 (PMC5382220; doi:10.3389/fmicb.2017.00602)
Supplement: Supplementary file 1 [file Data_Sheet_1.pdf]

# Genomic and Transcriptomic Insights into Calcium Carbonate Biomineralization by Marine Actinobacterium *Brevibacterium linens* BS258

*Yuying Zhu[1,2,3*], Ning Ma[1,2,3*], Weihua Jin[4], Shimei Wu[5], Chaomin Sun[1,2#]*

[1]*Key Laboratory of Experimental Marine Biology, Institute of Oceanology, Chinese Academy of Sciences, Qingdao, China*
[2]*Laboratory for Marine Biology and Biotechnology, Qingdao National Laboratory for Marine Science and Technology, Qingdao, China*
[3]*University of Chinese Academy of Sciences, Beijing, China*
[4]*College of Biotechnology and Bioengineering, Zhejiang University of Technology, Hangzhou, China*
[5]*College of Life Sciences, Qingdao University, Qingdao, China*

[#]*Address correspondence to Chaomin Sun*
*Tel.: +86 532 82898857; fax: +86 532 82898857.*
*E-mail address: sunchaomin@qdio.ac.cn*

[*]*These authors contributed equally to this work.*

## MATERIALS AND METHODS

**FTIR Analysis of Biominerals Produced by *B. linens* BS258**.

For FTIR, samples, standard $CaCO_3$ and KBr were dried in an oven at 105 °C overnight. The test sample was continuously ground to a fine powder for about 3 min using an agate mortar and pestle (Li et al., 2015). The samples were pressed into KBr pellets, and spectra were recorded over the region 4000-400 $cm^{-1}$ on a Nicolet-360 FTIR spectrometer (36 scans, at a resolution of 6 $cm^{-1}$) purged with $CO_2$-free dry air. A KBr pellet was used as a background control. The results were analyzed using OMNIC 8.0 and ORIGIN 8.0 program.

**Genome Sequencing and Assembly of *B. linens* BS258.**

The whole genome of *B. linens* BS258 was sequenced by PacBio single molecule real time sequencing technology with a 10 kb SMRTbell library. A total of 92 575 long reads (1 330 998 188 bp) were obtained where the N50 Read Length was 20 542 bp. Reads were assembled using SMRT Analysis 2.3.0. Coding genes were annotated by Prokaryotic Genome Annotation Pipeline (PGAP) version 2.10 software on NCBI (http://www.ncbi.nlm.nih. gov/genome/annotation prok/) and Rapid Annotation using Sub-system Technology (RAST: http://rast.nmpdr.org/) server (Aziz et al., 2008). Additional functional annotation was performed with 5 databases, they are KEGG (Kyoto Encyclopedia of Genes and Genomes) (Kanehisa et al., 2006), COG (Cluster of Orthologous Groups) (Tatusov et al., 2003), NR (Non-redundant Protein Database), GO (Gene Ontology) (Ashburner et al., 2000) and Swiss-Prot (Magrane and Consortium, 2011). The tRNA genes and rRNA genes were annotated with tRNAscan-SE v1.23 (Schattner et al., 2005) and rRNAmmer 1.2 (Lagesen et al., 2007), respectively.

**Transcriptional Profiling of Calcification of *B. linens* BS258.**

**Library Preparation for Strand-specific Transcriptome Sequencing.**

A total amount of 3 μg RNA per sample was used as input material for the RNA sample preparations. Sequencing libraries were generated using NEBNext® Ultra™ Directional RNA Library Prep Kit for Illumina® (NEB, USA) following manufacturer's recommendations and index codes were added to attribute sequences to each sample. rRNA is removed using a specialized kit that leaves the mRNA. Fragmentation was carried out using divalent cations under elevated temperature in NEBNext First Strand Synthesis Reaction Buffer（5X）. First strand cDNA was synthesized using random hexamer primer and M-MuLV Reverse Transcriptase（$RNaseH^-$）. Second strand cDNA synthesis was subsequently performed using DNA Polymerase I and RNase H. In the reaction buffer, dNTPs with dTTP were replaced by dUTP. Remaining overhangs were converted into blunt ends via exonuclease/polymerase activities. After adenylation of 3' ends of DNA fragments, NEBNext Adaptor with hairpin loop structure were ligated to prepare for hybridization. In order to select cDNA fragments of preferentially 150~200 bp in length, the library fragments were purified with AMPure XP system (Beckman Coulter, Beverly, USA). Then 3 μl USER Enzyme (NEB，USA) was used with

size-selected, adaptor-ligated cDNA at 37 °C for 15 min followed by 5 min at 95 °C before PCR. Then PCR was performed with Phusion High-Fidelity DNA polymerase, Universal PCR primers and Index (X) Primer. At last, products were purified (AMPure XP system) and library quality was assessed on the Agilent Bioanalyzer 2100 system.

**(1) Clustering and Sequencing (Novogene Experimental Department).**

The clustering of the index-coded samples was performed on a cBot Cluster Generation System using TruSeq PE Cluster Kit v3-cBot-HS (Illumia) according to the manufacturer's instructions. After cluster generation, the library preparations were sequenced on an Illumina Hiseq platform and paired-end reads were generated.

**(2) Data Analysis**

Raw data (raw reads) of fastq format were firstly processed through in-house perl scripts. In this step, clean data (clean reads) were obtained by removing reads containing adapter, reads containing ploy-N and low quality reads from raw data. At the same time, Q20, Q30 and GC content the clean data were calculated. All the downstream analyses were based on the clean data with high quality. Reference genome and gene model annotation files were downloaded from genome website directly. Both building index of reference genome and aligning clean reads to reference genome were used Bowtie2-2.2.3 (Langmead and Salzberg, 2012). HTSeq v0.6.1 was used to count the reads numbers mapped to each gene. And then FPKM of each gene was calculated based on the length of the gene and reads count mapped to this gene. FPKM, expected number of Fragments Per Kilobase of transcript sequence per Millions base pairs sequenced, considers the effect of sequencing depth and gene length for the reads count at the same time, and is currently the most commonly used method for estimating gene expression levels (Trapnell et al., 2009).

**(3) Differential Expression Analysis**

(For DESeq with biological replicates) Differential expression analysis of two conditions/groups (two biological replicates per condition) was performed using the DESeq R package (1.18.0) (Anders and Huber, 2010). DESeq provide statistical routines for determining differential expression in digital gene expression data using a model based on the negative binomial distribution. The resulting P-values were adjusted using the Benjamini and Hochberg's approach for controlling the false discovery rate. Genes with an adjusted P-value <0.05 found by DESeq were assigned as differentially expressed. (For DEGSeq without biological replicates) Prior to differential gene expression analysis, for each sequenced library, the read counts were adjusted by edgeR program package through one scaling normalized factor. Differential expression analysis of two conditions was performed using the DEGSeq R package (1.20.0) (Wang et al., 2010). The P values were adjusted using the Benjamini & Hochberg method. Corrected P-value of 0.005 and log2 (Fold change) of 1 were set as the threshold for significantly differential expression.

**(4) GO and KEGG Enrichment Analysis of Differentially Expressed Genes**

Gene Ontology (GO) enrichment analysis of differentially expressed genes was implemented by the GOseq R package, in which gene length bias was corrected(Young et al., 2010). GO terms with corrected P value less than 0.05 were

considered significantly enriched by differential expressed genes. KEGG is a database resource for understanding high-level functions and utilities of the biological system, such as the cell, the organism and the ecosystem, from molecular-level information, especially large-scale molecular datasets generated by genome sequencing and other high-through put experimental technologies (http://www.genome.jp/kegg/) (Kanehisa et al., 2008). We used KOBAS software to test the statistical enrichment of differential expression genes in KEGG pathways.

## REFERENCES


Anders, S., and Huber, W. (2010). Differential expression analysis for sequence count data. *Genome Biol.* 11. doi: 10.1186/gb-2010-11-10-r106

Ashburner, M., Ball, C.A., Blake, J.A., Botstein, D., Butler, H., Cherry, J.M., et al. (2000). Gene Ontology: tool for the unification of biology. *Nat. Genet.* 25, 25-29.

Aziz, R.K., Bartels, D., Best, A.A., DeJongh, M., Disz, T., Edwards, R.A., et al. (2008). The RAST server: Rapid annotations using subsystems technology. *Bmc. Genomics* 9. doi: 10.1186/1471-2164-9-75

Kanehisa, M., Araki, M., Goto, S., Hattori, M., Hirakawa, M., Itoh, M., et al. (2008). KEGG for linking genomes to life and the environment. *Nucleic Acids Res.* 36, D480-D484. doi: 10.1093/nar/gkm882

Kanehisa, M., Goto, S., Hattori, M., Aoki-Kinoshita, K.F., Itoh, M., Kawashima, S., et al. (2006). From genomics to chemical genomics: new developments in KEGG. *Nucleic Acids Res.* 34, D354-D357. doi: 10.1093/nar/gkj102

Lagesen, K., Hallin, P., Rodland, E.A., Staerfeldt, H.H., Rognes, T., and Ussery, D.W. (2007). RNAmmer: consistent and rapid annotation of ribosomal RNA genes. *Nucleic Acids Res.* 35, 3100-3108. doi: 10.1093/nar/gkm160

Langmead, B., and Salzberg, S.L. (2012). Fast gapped-read alignment with Bowtie 2. *Nat. Methods* 9, 357-U354. doi: 10.1038/Nmeth.1923

Li, Q.W., Csetenyi, L., Paton, G.I., and Gadd, G.M. (2015). $CaCO_3$ and $SrCO_3$ bioprecipitation by fungi isolated from calcareous soil. *Environ. Microbiol.* 17, 3082-3097.

Magrane, M., and Consortium, U. (2011). UniProt Knowledgebase: a hub of integrated protein data. *Database-the Journal of Biological Databases and Curation*. doi: 10.1093/database/bar009

Schattner, P., Brooks, A.N., and Lowe, T.M. (2005). The tRNAscan-SE, snoscan and snoGPS web servers for the detection of tRNAs and snoRNAs. *Nucleic Acids Res.* 33, W686-W689. doi: 10.1093/nar/gki366

Tatusov, R.L., Fedorova, N.D., Jackson, J.D., Jacobs, A.R., Kiryutin, B., Koonin, E.V., et al. (2003). The COG database: an updated version includes eukaryotes. *Bmc Bioinformatics* 4. doi: 10.1186/1471-2105-4-41

Trapnell, C., Pachter, L., and Salzberg, S.L. (2009). TopHat: discovering splice junctions with RNA-Seq. *Bioinformatics* 25, 1105-1111. doi: 10.1093/bioinformatics/btp120

Wang, L.K., Feng, Z.X., Wang, X., Wang, X.W., and Zhang, X.G. (2010). DEGseq: an R package for identifying differentially expressed genes from RNA-seq data. *Bioinformatics* 26, 136-138. doi: 10.1093/bioinformatics/btp612

Young, M.D., Wakefield, M.J., Smyth, G.K., and Oshlack, A. (2010). Gene ontology analysis for RNA-seq: accounting for selection bias. *Genome Biol.* 11. doi: 10.1186/gb-2010-11-2-r14

# RESULTS

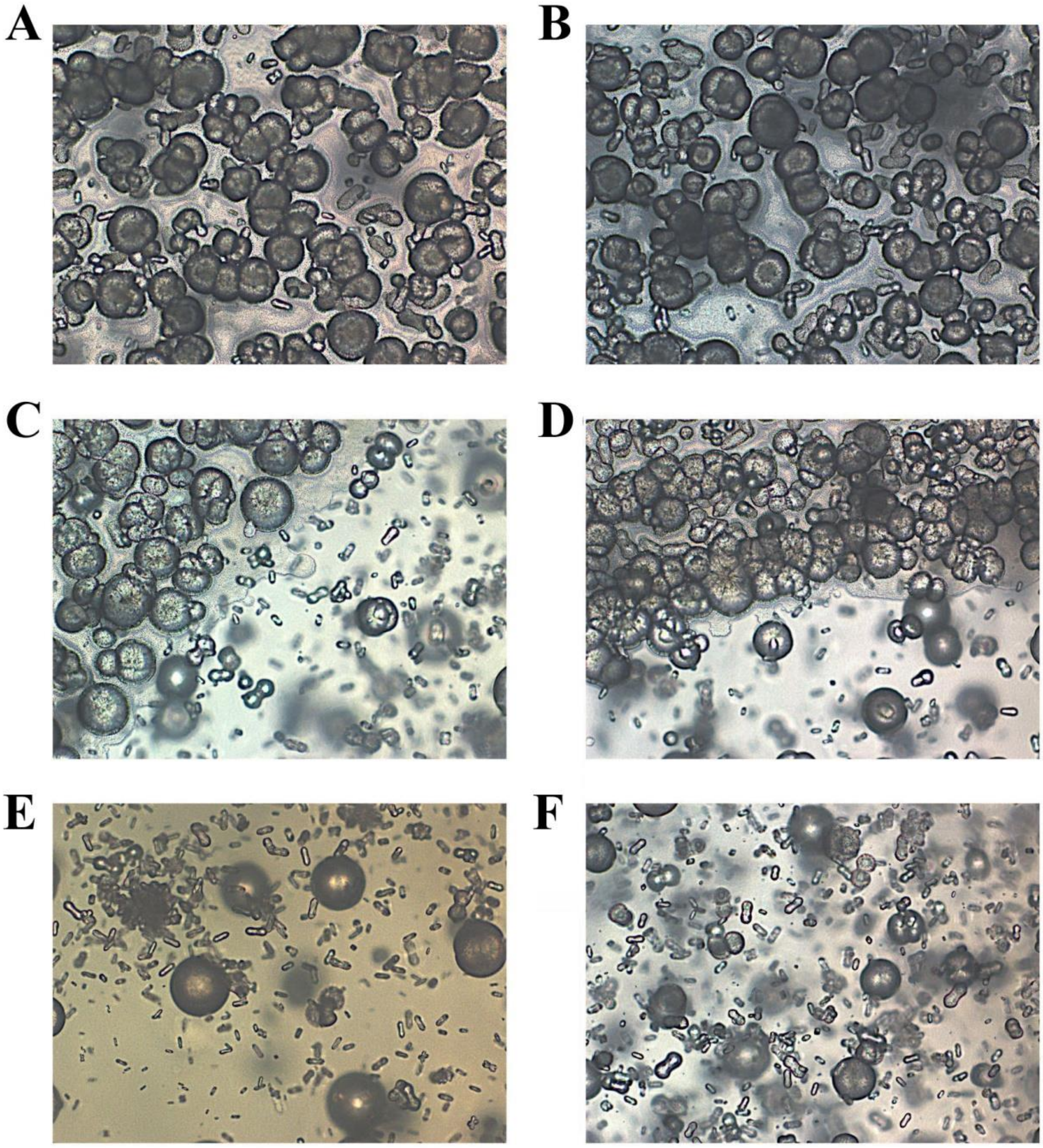


**FIGURE S1. Light micrographs of calcium carbonate crystals on the surface of the colony (A, B), around the colony (C, D) and far from the colony (E, F) precipitated by *B. linens* BS258.** All the pictures were taken with inverted microscope (Nikon TS100, Japan) at 200 X.

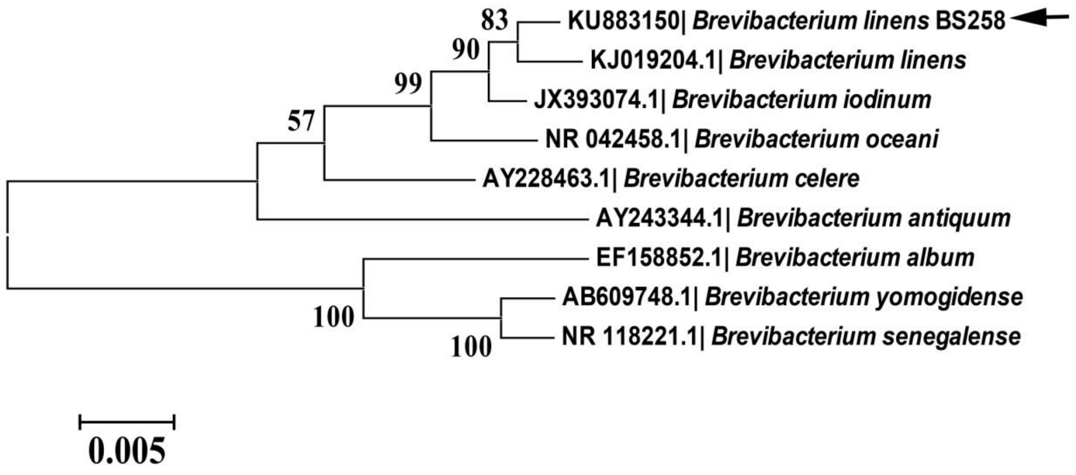


**FIGURE S2. The consensus phylogenetic tree of *B. linens* BS258 with other related strains obtained from GenBank (accession numbers are indicated before the species name) constructed by the neighbor-joining method.** Numbers above the branches are bootstrap values based on 1000 replicates.

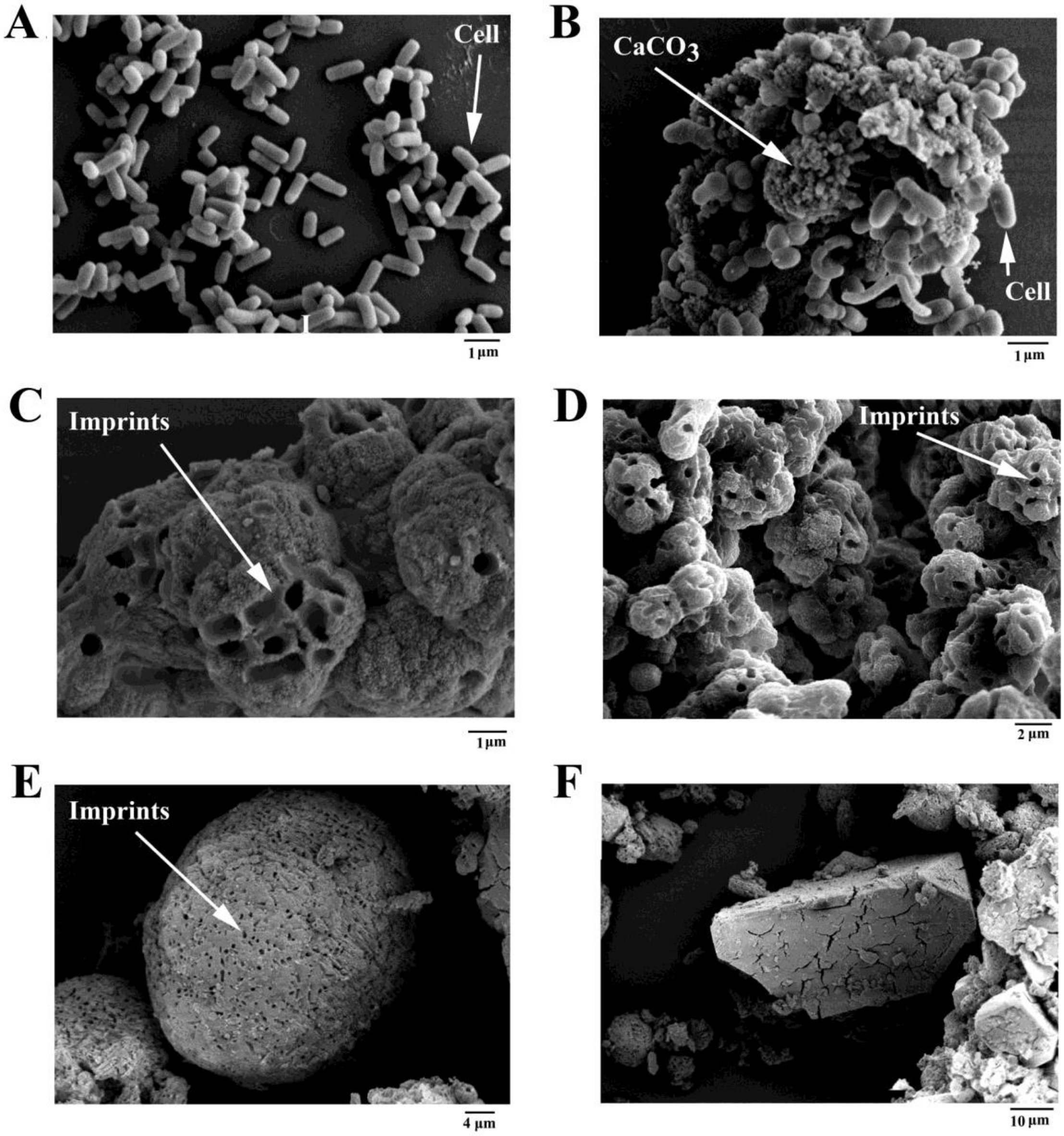


**FIGURE S3. Scanning electron micrographs of calcium carbonate crystals precipitated by *B. linens* BS258 in the 9219 base liquid media supplemented without (A) or with 30 mM $CaCl_2$ (B-F).** Typical images are shown from many similar examples.

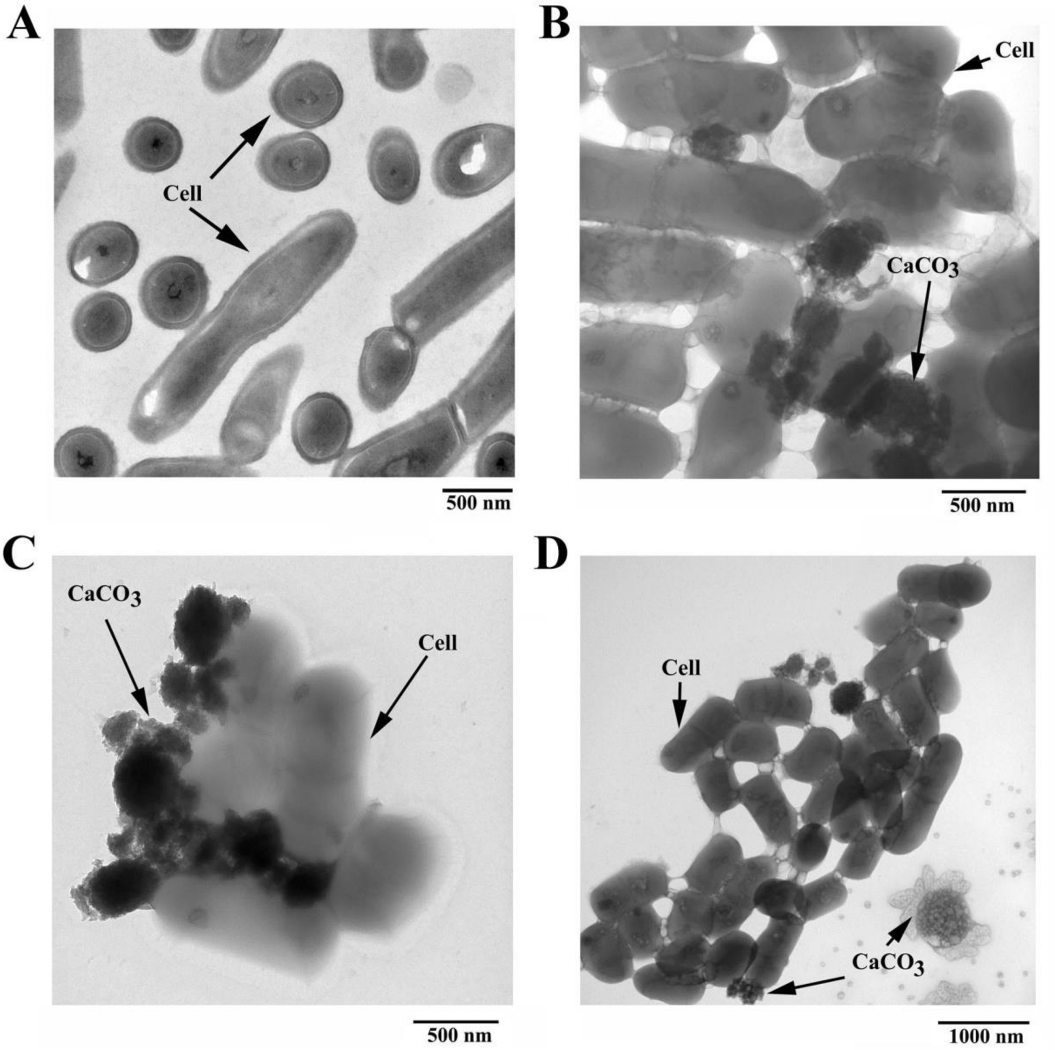


**FIGURE S4. Transmission electron micrographs of calcium carbonate crystals precipitated by *B. linens* BS258 in the 9219 base liquid media supplemented without (A) or with 30 mM $CaCl_2$ (B-D).** Typical images are shown from many similar examples.

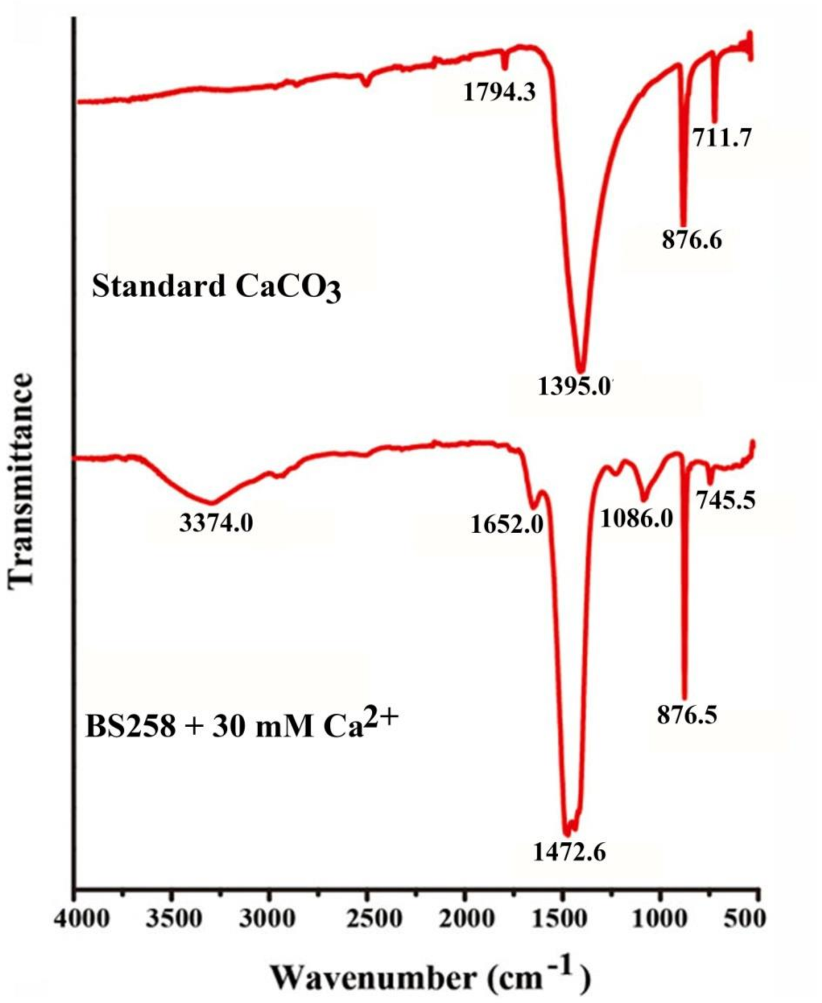


**FIGUE S5. Fourier transform infrared spectroscopy of minerals formed in 9219 base medium supplemented with 30 mM $CaCl_2$ after growth of *B. linens* BS258 for 10 days at 28 °C.** Typical spectra are shown from one of several determinations.

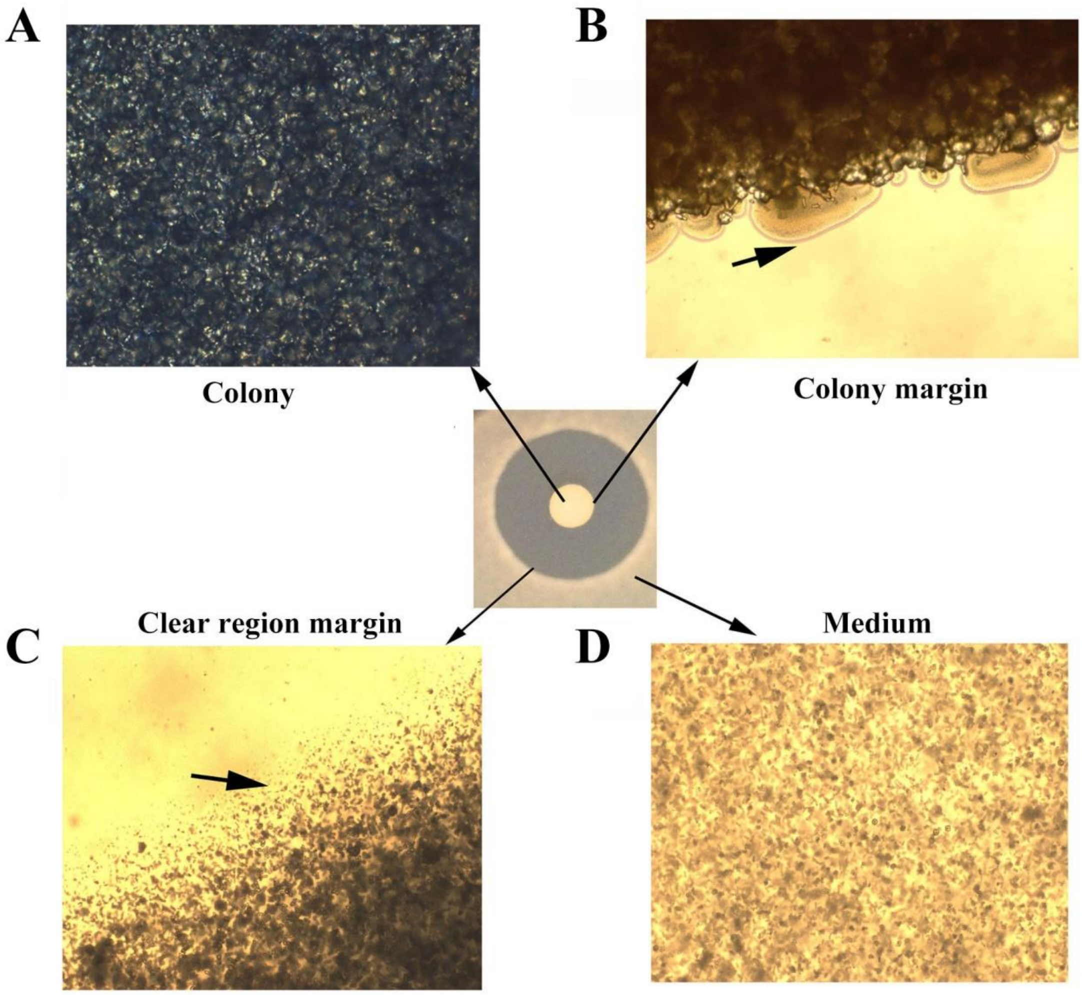


**FIGURE S6. Calcite precipitation or dissolution observation on the surface of the colony (A), around the colony margin (B), in the clear region margin (C) and in the media area far from the colony (D) in the $CaCO_3$-producing media supplemented with additional 150 mM $CaCl_2$ after growth of *B. linens* BS258 for 5 days at 28 °C.** All the pictures were taken with inverted microscope (Nikon TS100, Japan) at 100 X and typical images are shown from many similar examples.

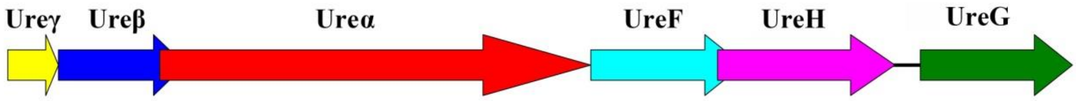


**FIGURE S7. The proposed urease (Ureγ, Ureβ, Ureα) and urease accessory proteins (UreF, UreH, UreG) encoding gene clusters of *B. linens* BS258 based on genome sequence analysis.** The accession numbers for the urease and urease accessory proteins gene clusters are as following, Ureγ (A2T55_02605), Ureβ (A2T55_02610), Ureα (A2T55_02615), UreF (A2T55_02620), UreH (A2T55_02625), UreG (A2T55_02630).

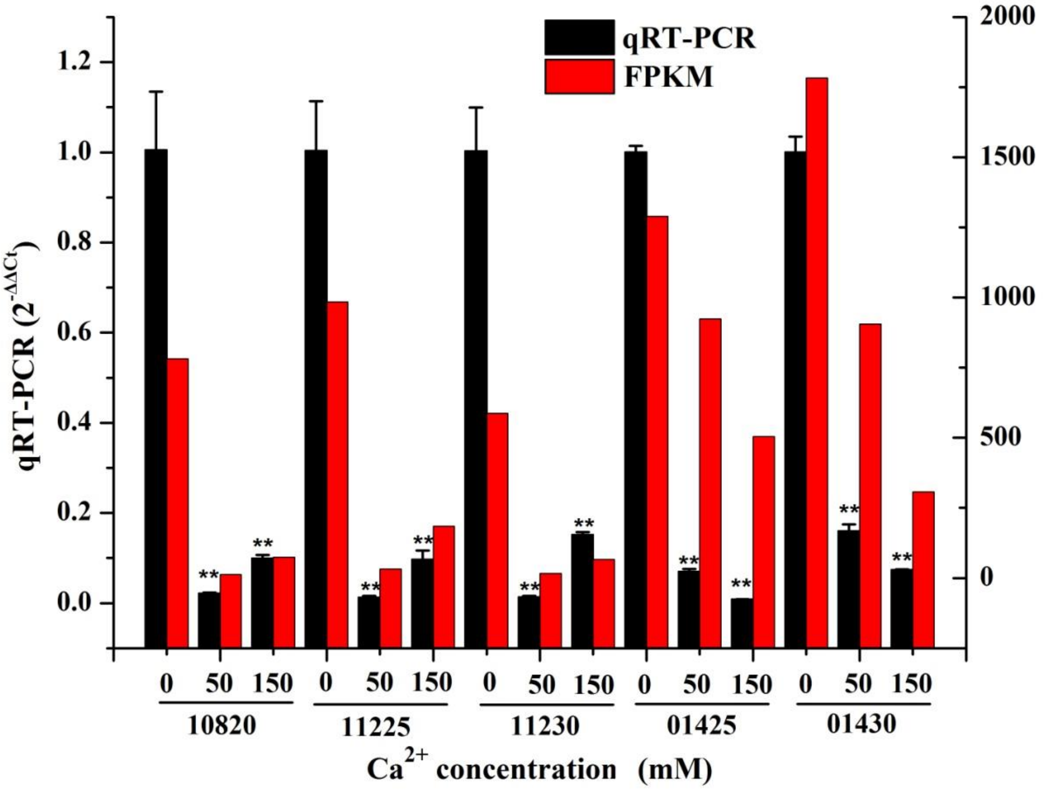


**FIGURE S8. The expression information of selected genes for qRT-PCR under different concentration of $Ca^{2+}$ stress condition.** The gene numbers (10820, 11225, 11230, 01425 and 01430) used for qRT-PCR were listed below the concentration of $Ca^{2+}$. FPKM: Fragmentsper Kilobase Million. The GenBank accession numbers for these five genes are A2T55_RS10820, A2T55_RS11225, A2T55_RS11230, A2T55_RS01425, A2T55_RS01430. *P* < 0.01 indicated by **.

**Table S1.** Primers for qRT-PCR used to check the expression of carbonic anhydrases.

| Primer name | Primer sequence |
|---|---|
| 16S rDNA-F | GCAGGGAAGAAGCGAAAGTG |
| 16S rDNA-R | CGTTTCCACAGCAGACGTGA |
| carbonic anhydrase1-F | TCGGCGATATGTTCGTCGTG |
| carbonic anhydrase1-R | AGTCTCACCCGAATCGTAGG |
| carbonic anhydrase2-F | GTGACGACGTTCCAGTTAGC |
| carbonic anhydrase2-R | GAGTTCGATGGGAGTGTCGAT |
| carbonic anhydrase3-F | ATGGTCATGAGCTGGCACTT |
| carbonic anhydrase3-R | CCCAGCGTGAAGTAGGAGTA |

**Table S2A.** Key genes' IDs and annotation under the KEGG term of "Ribosome".

| KEGG term | Gene ID | Annotation |
|---|---|---|
| Ribosome | A2T55_RS00590 | 30S ribosomal protein S18 |
| | A2T55_RS05785 | 50S ribosomal protein L11 |
| | A2T55_RS05790 | 50S ribosomal protein L1 |
| | A2T55_RS05900 | 30S ribosomal protein S12 |
| | A2T55_RS05905 | 30S ribosomal protein S7 |
| | A2T55_RS05925 | 50S ribosomal protein L3 |
| | A2T55_RS05930 | 50S ribosomal protein L4 |
| | A2T55_RS05935 | 50S ribosomal protein L23 |
| | A2T55_RS05940 | 50S ribosomal protein L2 |
| | A2T55_RS05945 | 30S ribosomal protein S19 |
| | A2T55_RS05950 | 50S ribosomal protein L22 |
| | A2T55_RS05965 | 50S ribosomal protein L29 |
| | A2T55_RS05975 | 50S ribosomal protein L14 |
| | A2T55_RS05980 | 50S ribosomal protein L24 |
| | A2T55_RS05985 | 50S ribosomal protein L5 |
| | A2T55_RS05990 | 30S ribosomal protein S8 |
| | A2T55_RS05995 | 50S ribosomal protein L6 |
| | A2T55_RS06000 | 50S ribosomal protein L18 |
| | A2T55_RS06010 | 50S ribosomal protein L30 |
| | A2T55_RS06055 | 50S ribosomal protein L36 |
| | A2T55_RS06060 | 30S ribosomal protein S13 |
| | A2T55_RS06065 | 30S ribosomal protein S11 |
| | A2T55_RS06340 | 50S ribosomal protein L25/general stress protein Ctc |
| | A2T55_RS09725 | 30S ribosomal protein S1 |
| | A2T55_RS10200 | 30S ribosomal protein S20 |
| | A2T55_RS11825 | 30S ribosomal protein S2 |
| | A2T55_RS11895 | 30S ribosomal protein S16 |
| | A2T55_RS11950 | 50S ribosomal protein L32 |
| | A2T55_RS13300 | 50S ribosomal protein L13 |
| | A2T55_RS16945 | 30S ribosomal protein S14 |
| | A2T55_RS16955 | 50S ribosomal protein L28 |

**Table S2B.** Key genes' IDs and annotation under the KEGG term of "Quorum sensing".

| KEGG term | Gene ID | Annotation |
|---|---|---|
| Quorum sensing | A2T55_RS05775 | preprotein translocase subunit SecE |
| | A2T55_RS07820 | peptide/nickel ABC transporter substrate-binding protein |
| | A2T55_RS07825 | peptide/nickel ABC transporter permease |
| | A2T55_RS07830 | peptide/nickel ABC transporter permease |
| | A2T55_RS07835 | peptide/nickel ABC transporter ATP-binding protein |
| | A2T55_RS10045 | preprotein translocase subunit YajC |
| | A2T55_RS10245 | long-chain fatty acid--CoA ligase |
| | A2T55_RS11900 | signal recognition particle protein |
| | A2T55_RS12310 | peptide/nickel ABC transporter ATP-binding protein |
| | A2T55_RS12315 | peptide/nickel ABC transporter permease |
| | A2T55_RS13810 | peptide/nickel ABC transporter permease |
| | A2T55_RS13815 | peptide/nickel ABC transporter permease |
| | A2T55_RS13820 | peptide/nickel ABC transporter substrate-binding protein |
| | A2T55_RS15105 | peptide/nickel ABC transporter ATP-binding protein |
| | A2T55_RS15110 | peptide/nickel ABC transporter permease |
| | A2T55_RS15115 | peptide/nickel ABC transporter permease |
| | A2T55_RS16225 | peptide/nickel ABC transporter ATP-binding protein |
| | A2T55_RS16230 | peptide/nickel ABC transporter permease |
| | A2T55_RS16235 | peptide/nickel ABC transporter permease |

**Table S2C.** Key genes' IDs and annotation under the KEGG term of "ABC transporter".

| KEGG term | Gene ID | Annotation |
|---|---|---|
| ABC transporter | A2T55_RS00315 | iron complex transport system substrate-binding protein |
| | A2T55_RS02580 | iron ABC transporter permease |
| | A2T55_RS03660 | spermidine/putrescine ABC transporter permease |
| | A2T55_RS03665 | spermidine/putrescine ABC transporter substrate-binding protein |
| | A2T55_RS03670 | spermidine/putrescine ABC transporter ATP-binding protein |
| | A2T55_RS04715 | iron ABC transporter substrate-binding protein |
| | A2T55_RS06575 | ABC transporter permease |
| | A2T55_RS06580 | $Fe^{3+}$-hydroxamate ABC transporter |
| | A2T55_RS07465 | cobalt transporter |
| | A2T55_RS07580 | ABC transporter substrate-binding protein |
| | A2T55_RS07610 | ABC transporter permease |
| | A2T55_RS07615 | iron ABC transporter permease |
| | A2T55_RS07620 | iron-dicitrate transporter ATP-binding subunit |
| | A2T55_RS08705 | molybdate ABC transporter substrate-binding protein |
| | A2T55_RS08710 | molybdenum ABC transporter ATP-binding protein |
| | A2T55_RS08725 | ABC transporter substrate-binding protein |
| | A2T55_RS08730 | amino acid ABC transporter permease |
| | A2T55_RS08735 | amino acid ABC transporter permease |
| | A2T55_RS11985 | ectoine/hydroxyectoine ABC transporter permease subunit EhuC |
| | A2T55_RS12935 | cell division protein FtsX |
| | A2T55_RS12940 | cell division ATP-binding protein FtsE |
| | A2T55_RS13140 | sugar ABC transporter permease |
| | A2T55_RS13145 | sugar ABC transporter permease |
| | A2T55_RS13150 | sugar ABC transporter substrate-binding protein |
| | A2T55_RS13165 | sugar ABC transporter ATP-binding protein |
| | A2T55_RS13655 | methionine ABC transporter ATP-binding protein |
| | A2T55_RS13660 | metal ABC transporter permease |
| | A2T55_RS13665 | ABC transporter |
| | A2T55_RS16895 | hypothetical protein/molybdate transport system ATP-binding protein |

**Table S3.** Primers for qRT-PCR used to verify the transcriptome results.

| Primer name | Primer sequence |
|---|---|
| 16S rDNA-F | GCAGGGAAGAAGCGAAAGTG |
| 16S rDNA-R | CGTTTCCACAGCAGACGTGA |
| A2T55_RS10820-F | GGTCGGTCTCGAAATGGTCA |
| A2T55_RS10820-R | TGGACGGTGCGTATTTGTCA |
| A2T55_RS11225-F | GAACCTGCTCGTCAAACCCA |
| A2T55_RS11225-R | GCTCTGCCAGAAGCTGTGAT |
| A2T55_RS11230-F | CCGACATACCAGGCAGTGTTCA |
| A2T55_RS11230-R | TGCTGTTCCTCGTAGTCACCCT |
| A2T55_RS01425-F | CCGCTGTATGAGGTCTTCGT |
| A2T55_RS01425-R | AACATCGGGTCCTTCTCGTC |
| A2T55_RS01430-F | CGTCAAGGAGATGTCCTACCA |
| A2T55_RS01430-R | GTCTCGAAGAGTTCGGACCA |
